# Family nursing with the assistance of network improves clinical outcome and life quality in patients underwent coronary artery bypass grafting: A consolidated standards of reporting trials-compliant randomized controlled trial

**DOI:** 10.1097/MD.0000000000023488

**Published:** 2020-12-11

**Authors:** Liying Jin, Ruijin Pan, Lihua Huang, Haixia Zhang, Mi Jiang, Hao Zhao

**Affiliations:** Department of Cardiac Surgery, The First Hospital of Jilin University, Changchun, China.

**Keywords:** coronary artery bypass grafting, family nursing through network, heart disease, life quality, outcome

## Abstract

**Background:**

Family nursing with the assistance of network (FNAN) improves nurses’ practice and provides family/community-oriented nursing care. This study aimed to explore the effects of FNAN on the clinical outcome and life quality of coronary atherosclerotic heart disease (CHD) patients underwent coronary artery bypass grafting (CABG).

**Trial Design:**

This study is a randomized, placebo-controlled and double-blind trial.

**Methods:**

One-hundred and twelve patients underwent CABG were randomly divided into control group (CG, routine family nursing care) and experimental group (EG, FNAN) and the allocation ratio was 1:1. The situation of anxiety and depression were analyzed using the Hamilton Anxiety Scale (HAMA) scale and Hamilton Depression Scale (HAMD). Sleep quality was measured by using Pittsburgh Sleep Quality Index (PSQI). Lung function parameters were measured, including minute ventilation (MVV), partial pressure of oxygen (PaO_2_), partial pressure of arterial carbon dioxide (PaCO_2_), oxygen saturation measurement by pulse oximetry (SpO_2_), forced expiratory volume in 1 second (FEV1) and forced vital capacity (FVC). Life quality was measured by using Chronic Obstructive Pulmonary Disease Assessment Test (CAT).

**Results:**

After a 3-month intervention, 10 and 6 patients were lost in the CG and EG groups, respectively. The scores of HAMA, HAMD, PSQI and CAT were reduced in the EG group when compared with the CG group (*P* < .05). The values of MVV, PaO_2_, SpO_2_, FEV1 and FVC in the EG group was higher than those in the CG group whereas the levels of PaCO_2_ in the EG group was lower than those in the CG group (*P* < .05). PSQI score had a strong relationship with the values of MVV, PaO_2_, PaCO_2_, SpO_2_, FEV1, and FVC.

**Conclusion:**

FNAN improves the clinical outcome and life quality in the patients underwent CABG.

## 1 Introduction

According to the World Health Organization (WHO), coronary heart disease (CHD), as the “first killer,” is the most common cause of human death.^[1]^ With the changes in people's living standards and habits, CHD incidence is increasing and accounts for more than 20% in all mortality rates.^[2]^ Coronary arterial bypass grafting (CABG) is an important surgical treatment for CHD patients.^[3]^ Previous studies have reported that the patients receiving CABG can achieve longer survival than interventional therapy.^[4]^ CABG is a relatively complicated operation often with large trauma wounds. The patients often have great fear and mental stress because serious complications may occur after CABG operation, which may endanger the patient's life. Therefore, CABG surgery has undoubtedly increased the mental and psychological stress. On the other hand, pulmonary abnormalities often follow CABG operation.^[5]^ Cognitive dysfunction, depression, and anxiety are also major concerns in the patients undergoing CABG.^[6]^

Nursing is very critical to protect mental and physical health.^[7]^ Family nursing is considered to be a feasible intervention in the routine care offered in heart failure patients.^[8]^ However, the pathogenesis of CHD is complex and its development cannot be precisely defined and predicted^[9,10]^ while the individual nurse has limited knowledge about the CHD prevention and treatment programs and is limited in basic nursing education programs and personal skills. Furthermore, the patients may under- or overreport the presence of their medical situation because of unintended incentives or the hope for the benefits associated with a diagnosis,^[11]^ which will increase the difficulty of nursing care. Even worse, the nurse faculty shortage is still the main issue in nursing practice or health care.^[12]^ Therefore, it is necessary to explore the new nursing method to provide continuous clinical nursing care and multiple care arrangements to the CHD patients to avoid complications whenever possible, thereby improving their clinical outcomes.^[13]^ Multidisciplinary nursing care is beneficial to the rehabilitation of the patient's lung function.^[14,15]^

Network development promotes joint and collaborative evaluations of multidisciplinary nursing care. A practitioner can learn more comprehensive nursing knowledge about family nursing with the assistance of network (FNAN) platform to improve nursing care service,^[16]^ which including the newest research findings, the clinical nursing experience, the patients’ requirement, and nursing resources.^[17]^ Meanwhile, the nurses can contact patients via a Wi-Fi network,^[18]^ which is continuously running and visible in the hospital, home and community in different ways. Different nurses and patients can share knowledge and successful experiences of nursing and improve multidisciplinary nursing care via the network. The network was managed by our hospital and hosted by a nurse leader. FNAN may exert many health-promoting properties and improve depression, sleep quality, anxiety and life quality.^[19]^ The study aimed to investigate the effect of FNAN on clinical outcomes in the patients undergoing CABG.

## 2 Materials and methods

### 2.1 Participants

This is a randomized single-blind controlled clinical trial. Before the study, all procedures were approved by the Ethics Committee of The First Hospital of Jilin University. The study participants were informed of the purpose, content, and procedures of the study before participating in the study. The confidentiality principle was fully followed involving privacy and personal safety. The patient information was used only for this study and cannot be used for any other purposes. The research results would help to improve the attention of medical staff to patients with sleep disorders, and also helped to develop a scientific and effective nursing intervention program for improving the mental situation and sleep quality of the patients who received CABG. From March 2017 to September 2018, the CHD patients, underwent CABG, were selected from our hospital

### 2.2 Selection criteria

Inclusion criteria included that the patients who met the New York Heart Association (NYHA) functional classification II and III;^[20]^ and the patients underwent CABG for stable coronary artery disease or acute coronary syndrome. Exclusion criteria included the previous history of mental illness and/or mental illness; disturbance of consciousness; communication disorders, without normal Chinese communications; pregnant; long-term application of sedative drugs and brain damage. The patients were unable to cooperate with nursing activity.

### 2.3 Sample size calculation

The sample size was calculated according to size effect and the two-sample mean comparison formula: $N=\frac{\left(q_{1}^{-1}+q_{2}^{-1}\right){\left(t_{\alpha /2}+t_{\beta }\right)}^{2}S^{2}}{\delta^{2}}$. A two-sided test was used, where class I error is a = 0.05, class II error is β=0.10, test power is 1-P = 0.90, and δ is the two populations. The difference between the numbers and the S is the standard deviation (S.D.) of the two samples. The literature review finally determines δ=3.53, S2 = 4.75, and qi∗q2 respectively indicates the distribution ratio of the sample content in the two groups. In this study, the two samples were in the same ratio, i.e. q1 = q2. = 0.5, t_α/2(∞)_ = t_0.05(∞)_ = 1.960 and t_β(∞)_ = t_0.10(∞)_ = 1.282. The sample size of each group should be no less than 45 cases. The current 112 patients allowed for a 20% loss to follow up while maintaining more than 80% power.

### 2.4 Patients grouping

The study was a randomized, double-blind, and parallel trial. Informed consent was obtained from patients who were eligible for the study after more than 1-month CABG. The patients were selected by 10 CHD experienced experts using the above 2.2. selection criteria and enrolled by the same experts. Allocation concealment (for patients, caregivers, and assessors) was performed to minimize selection bias and subsequent interference. The patients were randomized by means of a computer-generated allocation sequence in a 1:1 ratio to the FNAN group (EG) and the control group (CG). There was no restriction such as blocking and block size, though there was a balance between the 2 groups in size and baseline characteristics. Each subject entered his or her group by using the number in the sequentially numbered container to conceal the sequence until interventions were assigned. The assistants assigned the patients to their groups according to the numbers. In the end, there were 56 cases in the EG group (the patients received family nursing with the help of FNAN platform) and 56 cases in the CG group (the patients received normal family nursing). The patients and nurses were blinded to study group assignment. Thus, a total of 112 patients were included in the trial at our hospital. The data of the present experiment were collected by 4 nurses and analyzed by 4 statisticians at our hospital.

### 2.5 Intervention

From October 2018 to October 2019, family nursing was implemented in the both groups according to previously reported method with modification.^[21]^ In the CG group, regular family nursing care was performed:

(1) psychological nursing was carried out to alleviate anxiety.
(2) The patients were examined by electrocardiogram (ECG), observation, and mind recordings, heart rhythm and vital signs (including breathing, pulse, body temperature, and blood pressure).
(3) The nurses took practical actions of family nursing (focused on CHD development assessments, management of patients’ behavior and performance measures) to promote the health and prevent CHD development.
(4) Once the CHD aggregated, CHD doctors were told immediately and took effective measures immediately.

In the EG group, FNAN platform was used. A nurse leader with rich family nursing experiences and strong communication skills was chosen and 10 family nurses were selected to form an FNAN team. The team leader guided all members to collect data, design FNAN program and investigate the implementation of the program. All members of the group need to understand the purpose and specific process of FNAN program. The platform was established by inviting professionals to build an interactive platform online via a Wi-Fi network (a WeChat virtual community).^[22]^ The connection was performed by scanning the QR (Quick Response) code of the patient and the nurses. The platform also facilitated multiple communication among patients and nurses. The nurses contacted patients to encourage them to speak talk about their problems in group chats and share the progression in the rehabilitation process. In the group chat, the nurses provided family healthy guidance and advice to patients’ questions. The nurses determined the patient's condition, solved patient's doubts, exchanged their experiences, improved their negative emotions and promoted patient's cooperation for treatment. Most problems were solved by referring to the information form network platform when the nurses met some difficulties during family nursing. The platform provided the evidence-based professional consulting and supportive family nursing care based on technology that improved the efficiency of patients’ outcomes. The nurses analyzed all data uploaded by the patient, including physiological parameters, including heart rate, blood pressure, blood sugar, and ECG. Doctors and nurses can monitor patient health via the platform and thus reduced patient transportation time and increased the ability to offer immediate help in emergency situations. Patients are also able to consult their family nursing specialist via telecommunication to receive test results and obtain advice with a 24-h service. The platform provided around-the-clock family nursing care accessible from home promotes healthy living and reduces complications in CHD patients after CABG surgery.

Both groups were given diuretic, low-flow oxygen, tube expansion, asthma-preventing, expectorant, anti-infection and other symptomatic treatment.^[23]^ Persistent oxygen inhalation was given daily with the oxygen flow at 2 to 4 L/min.^[24]^ This study provided intervention details to allow replication and implementation by nurses and researchers for the exact study (Sup1).

### 2.6 Anxiety and depression, and sleep quality measurement

Anxiety and depression, and sleep quality are closely associated with life quality.^[25]^ Therefore, all these parameters were measured between the 2 groups. Anxiety and depression were measured by using Hamilton Depression Rating Scale (HAMD), Hamilton Anxiety Scale (HAMA). Sleep quality was measured by using Pittsburgh Sleep Quality Index (PSQI) and the scores more than 7 were regarded as poor sleep quality.^[26]^ Anxiety and depression, and sleep quality measurement was performed at our hospital clinic before and after 3-month family nursing.

### 2.7 Lung function measurement and evaluation criteria

Poor sleep quality often affects lung function, and lung function was measured using the following parameters.^[27,28]^ Chronic Obstructive Pulmonary Disease Assessment Test (CAT) was measured by using by St. George's respiratory disease questionnaire.^[29]^ Arterial blood gas analysis was performed by using a fully automatic blood gas analyzer (i-STAT 200A or 300F, i- STAT Co., East Windsor, NJ) at 37°C. Lung function parameters were measured, including MVV, minute ventilation; PaO_2_, partial pressure of oxygen; PaCO_2_, partial pressure of arterial carbon dioxide and SpO_2_, oxygen saturation measurement by pulse oximetry. Meanwhile, FEV1 (forced expiratory volume in 1 second) was measured by an electronic spirometer (Wright Ventilometer, Clement Clarke International, London) and FVC (forced vital capacity) was measured by using Vitalograph-Compact II (Vitalograph Medical Instruments, Buckingham). Lung function was measured at our hospital clinic before and after 3-month family nursing (Sup1).

The efficacy evaluation criteria were formulated as follows: significant effect, good postoperative recovery without complications and complete recovery of respiratory function; effective, no complications occurred and respiratory function was improved. Invalid, patients with respiratory diseases after surgery. Total effective rate = (significant effect + effective)/total number of cases × 100%.

### 2.8 Primary and secondary outcome

Primary outcomes were measured after a 10-day intervention, including HAMD, HAMA, PSQI and CAT scores. Secondary outcomes were measured after 3-month intervention, including the changes of depression and anxiety, sleep quality, and lung function in the patients underwent CABG (Sup1).

### 2.9 Statistical analysis

All data were presented as ± S.D. Excel 2016 was used to establish a database and SPSS21.0 software was used for statistical analysis of patient's general data (primary outcomes and secondary outcomes). Before and after a 3-month FNAN intervention, lung function parameters between the 2 groups were analyzed by 2 independent samples *t*-test. A Mann-Whitney *U* test between the 2 groups (primary outcomes and secondary outcomes) was used in accordance with the normal distribution (Sup1). Pearson correlation coefficient was used to explore the relation between PSQI scores and lung function parameters. All statistical tests were statistically significant at *P* < .05.

## 3 Results

### 3.1 Baseline characteristics

**Figure 1:**
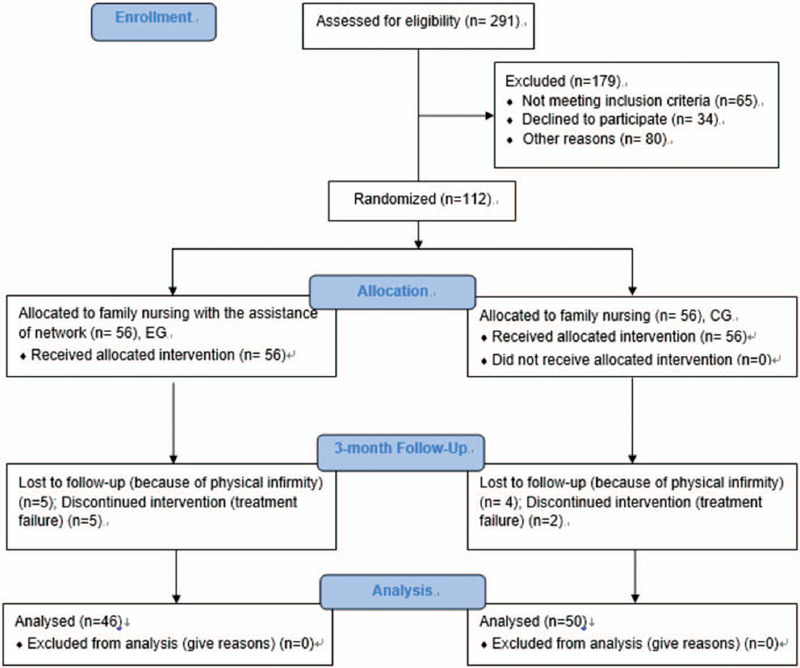
The CONSORT flow diagram. EG, FNAN intervention group and CG, common-care group. Intervention duration was 3 months.

The recruitment period was from March 2017 to September 2018, the CHD patients underwent CABG, were selected from our hospital. From October 2018 to October 2019, family nursing was implemented in the both groups and the patients were follow-up (Sup1). After a 3-month nursing care intervention, 10 and 6 patients (their data were not complete) were lost from the CG and EG groups, respectively. Therefore, a total of 96 patients finished the whole experiment (Fig. 1 and Sup1). The statistical difference for the baseline characteristics (gender, age, educational levels, coronary lesions, degree of pulmonary ventilatory dysfunction, NYHA and APACHE II classification so on) were insignificant between the 2 groups (Table 1, *P* > .05).

**Table 1:** Baseline characteristics between two groups.

Parameters	CG (n = 46)	EG (n = 50)	χ2 or t values	*P* values
Gender, male (%)	32 (69.56)	30 (60.00)	0.479	.489
Education levels				
Junior high school and below	4 (8.69)	6 (12.00)	0.327	.849
High school and specialist	26 (56.52)	30 (60.00)		
Bachelor degree or above	16 (34.78)	14 (28.00)		
Monthly salary, RMB				
<2000	6 (13.04)	8 (16.00)	0.671	.715
2000∼5000	22 (47.82)	28 (56.00)		
>5000	18 (39.13)	14 (28.00)		
Payment of medical treatment				
Self-pay	4 (8.69)	6 (12.00)	0.814	.846
Employee medical insurance	14 (30.43)	20 (40.00)		
Cooperative medical care	26 (56.52)	22 (44.00)		
other	2 (4.34)	2 (4.00)		
Coronary lesion				
Mono-vascular lesion	4 (8.69)	6 (12.00)	0.169	.918
Bifurcation Lesions	18 (39.14)	20 (40.00)		
Trifurcation Lesions	24 (52.17)	24 (48.00)		
Age	45.30 ± 7.31	43.76 ± 6.57	0.77	.45
APACHE II	12.3 ± 51.89	11.68 ± 1.61	1.32	.19
NYHA classification				
II	26 (56.52)	30 (60.00)	0.119	.730
III	20 (43.48)	20 (40.00)		
Degree of pulmonary ventilatory dysfunction				
Mild	26 (56.52)	28 (56.00)	0.041	.979
Moderate	14 (30.43)	16 (32.00)		
Severe	6 (13.05)	6 (12.00)		

NYHA = New York Heart Association and APACHE II, a severity-of-disease classification system.

### 3.2 FNAN intervention increased therapeutic rates of CHD patients

Therapeutic rate was evaluated based on the complications and complete recovery of respiratory function. After a 3-month intervention, the total effective rate of treatment in the EG group was 96.0%, which was higher than 41.3% in the CG group. The difference was statistically significant (*P* < .05, Table 2). The result suggested that FNAN intervention increased the therapeutic rates of CHD patients when compared with the patients only received routine nursing care.

**Table 2:** The effect of FNAN on the therapeutic rates of CHD patients.

Parameters	CG (n = 46)	EG (n = 50)	χ2	*P*
Significant effective, cases (%)	4 (8.7)	38 (76.0)	49.99	< .001
Effective, cases (%)	15 (32.6)	10 (20.0)		
Invalid, cases (%)	27 (58.7)	2 (4)		

The statistical difference was significant if *P* < .05. CHD = coronary atherosclerotic heart disease, FNAN = family nursing with the assistance of network.

### 3.3 Primary outcomes

**Figure 2:**
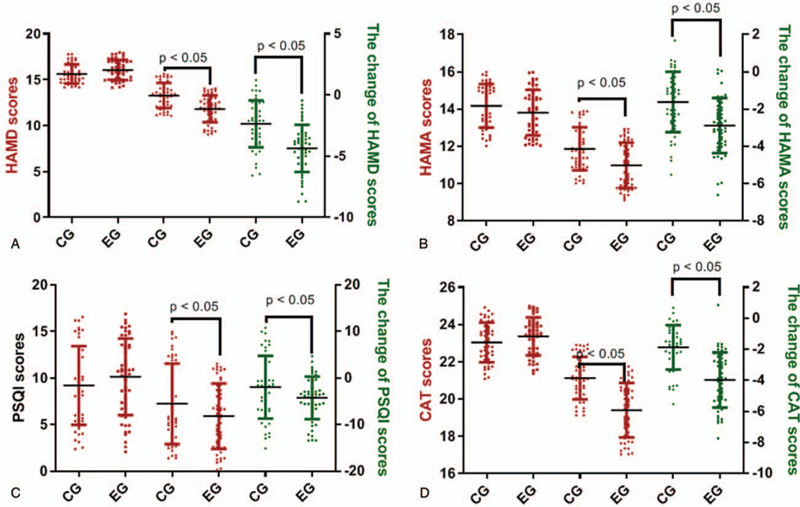
The effects of FNAN on depression, anxiety, sleep quality and life quality in CHD patients. A, HAMD scores; B, HAMA scores; C, PSQI scores; D, CAT scores. ^∗^*P* < .05 vs the CG group.

In the primary outcomes, there were 56 cases in the EG group (the patients received family nursing with the help of FNAN platform) and 56 cases in the CG group (the patients received normal family nursing). All of them received intended treatment, and were analyzed for the primary outcome (Sup1). Before and after the 10-day intervention, the statistical differences were insignificant for the scores of HAMD (Fig. 2A), HAMA (Fig. 2B), PSQI (Fig. 2C) and CAT (Fig. 2D) between the 2 groups (*P* > .05). The results suggested that a short-term intervention of FNAN could not affect the mental situation, sleep quality and lung function in the patients.

### 3.4 FNAN intervention reduced the incidence of complications in CHD patients

The incidence of complications in the EG group was 10.0%, which was lower than 32.6% in the CG group. The difference was statistically significant (Table 3, *P* < .05). The results suggested that FNAN intervention reduced the incidence of complications in CHD patients when compared with the routine family nursing in the CG group.

**Table 3:** The effect of FNAN on complications in the CHD patients.

Parameters	CG (n = 46)	EG (n = 50)	χ2	*P*
Respiratory system infection, cases (%)	6 (13.0)	2 (4.0)	7.42	.006
Lung infection, cases (%)	4 (8.7)	2 (4.0)		
Blocked breathing, cases (%)	5 (10.9)	1 (2.0)		

The statistical difference was significant if *P* < .05. CHD = coronary atherosclerotic heart disease, FNAN = family nursing with the assistance of network

### 3.5 FNAN improved mental health disorders and lung function of CHD patients better than the control group

After the 3-month intervention, the scores of HAMD (Fig. 2A), HAMA (Fig. 2B), PSQI (Fig. 2C) and CAT (Fig. 2D) were reduced significantly in the 2 groups (*P* < .05). Raw data were provided in Sup2 file. The reduction in the EG group was more than that in the CG group (Fig. 2, *P* < .05). The results suggested that FNAN improved mental health disorders (depression and anxiety) and lung function in the CHD patients better than that in the control group.

### 3.6 FNAN intervention improved lung function parameters

**Figure 3:**
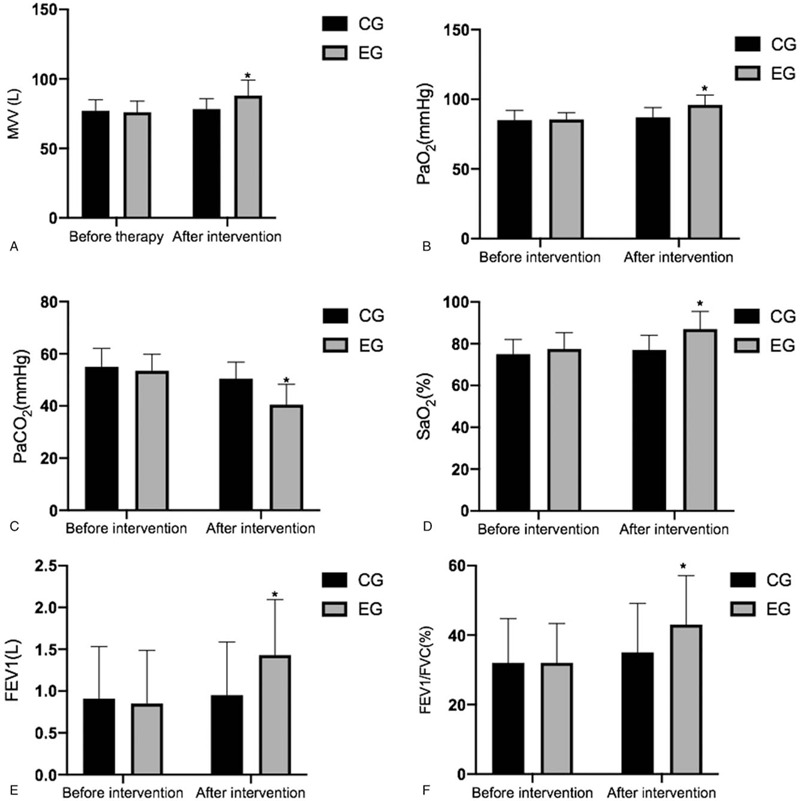
The effects of FNAN on lung function parameters in CHD patients. A, MVV, minute ventilation; B, PaO_2_, partial pressure of oxygen; C, PaCO_2_, partial pressure of arterial carbon dioxide; D, SpO_2_, oxygen saturation measurement by pulse oximetry; E, FEV1, forced expiratory volume in 1 second; F, FEV1/FVC (forced vital capacity). ^∗^*P* < .05 vs the CG group.

The statistical difference for all lung function parameters was insignificant between the 2 groups before the intervention and after a 10-day intervention (*P* > .05), including MVV (Fig. 3A), PaO_2_ (Fig. 3B), PaCO_2_ (Fig. 3C), SpO_2_ (Fig. 3D), FEV1 (Fig. 3E), FVC (Fig. 3F) and FEV1/FVC (Fig. 3G). After a 3-month intervention, FNAN intervention improved the values of MVV (Fig. 3A), PaO_2_ (Fig. 3B), PaCO_2_ (Fig. 3C), SpO_2_ (Fig. 3D), FEV1 (Fig. 3E), and FEV1/FVC (Fig. 3F) in the EG group higher than those in the CG group (*P* < .05). Raw data were provided in Sup3 file. The results suggest that FNAN intervention improves lung function.

### 3.7 PSQI scores had a strong relationship with the parameters of lung function

**Figure 4:**
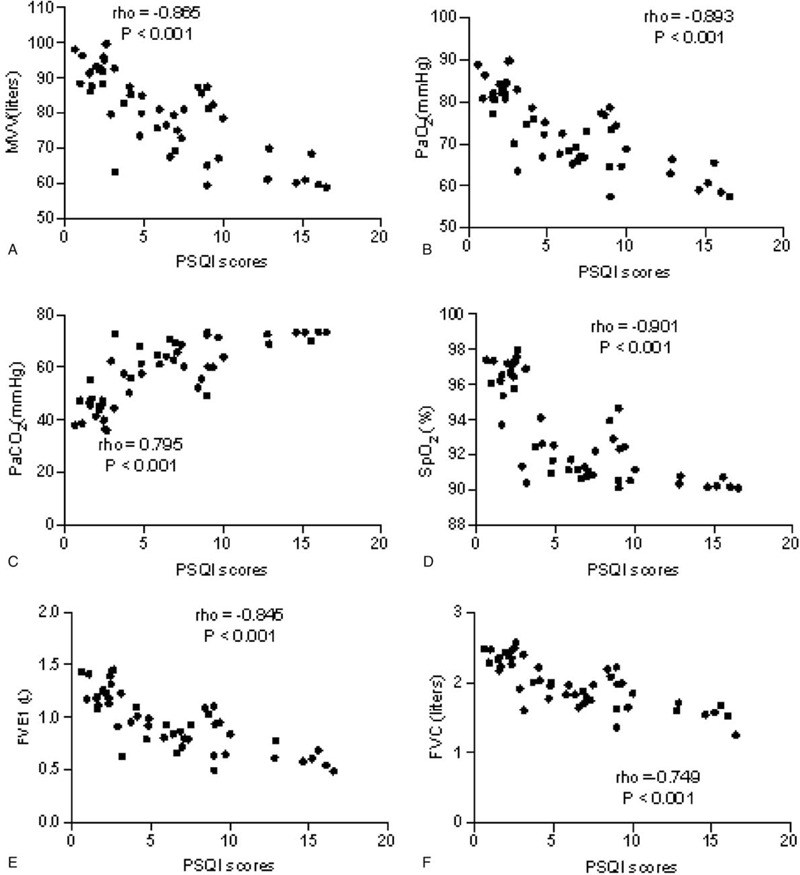
The correlation between PSQI scores and the value of lung function parameters in CHD patients. A, MVV, minute ventilation; B, PaO_2_, partial pressure of oxygen; C, PaCO_2_, partial pressure of arterial carbon dioxide; D, SpO_2_, oxygen saturation measurement by pulse oximetry; E, FEV1, forced expiratory volume in 1 second; F, FVC (forced vital capacity). There was a strong positive correlation if rho > 0.5 and or a strong negative correlation if rho < -0.5.

The correlation test showed that the decrease in PSQI scores resulted in the increase in the values of MVV (Fig. 4A), and PaO_2_ (Fig. 4B), the reduction in PaCO_2_ (Fig. 4C), and the increase in SpO_2_ (Fig. 4D), FEV1 (Fig. 4E), and FVC (Fig. 4F) (*P* < .001). All these results suggest that PSQI scores have a strong relationship with the parameters of lung function parameters. Sleep quality may be a predominant factor and possibly affects the lung function of CHD patients.

## 4 Discussion

The patient's FEVi values were significantly higher in the EG group than in the CG group after a 3-month intervention (Fig. 3E). The reason may be that the family nursing via FNAN platform reduced the severity of patients’ depression and anxiety, which possibly improved sleep quality (Fig. 2)^[30,31]^ and life quality. Comparatively, network nursing care has been reported to reduce cancer-related fatigue and improve the overall quality of life better than the routine nursing care in cancer patients.^[32]^ Lung function may also be related to clinical pain and physical weakness, so that the patient's respiratory movement was limited, resulting in abnormal breathing patterns, decreased tidal volume and effective ventilation, and impaired oxygenation.

The present study showed that the lung function parameters in the EG group were significantly improved when compared with those in the CG group after a 3-month intervention (Fig. 3). PSQI scores had a strong relationship with lung function parameters (Fig. 4). On the other hand, the nurses used progressive relaxation training to help patients to relax, and more likely to enter sleep. Meanwhile, FNAN improved therapeutic rates (Table 2). It is indicated that the nursing intervention based on the network is a potential approach to effectively improve mental health disorders, sleep quality and lung function parameters of patients. Clinics can be run by nurses, clinical officers and medical assistants, but most of them may be with limited knowledge of lung disease management. An established network of nursing staff will provide broad knowledge for better nursing care in the patients.^[33]^

The nurses in the CG group used routine nursing care and often ignored the changes in the patient's psychological mood. This may be related to the lack of multiple protective programs in the group. A multiple nursing intervention was performed in the EG group and the nurses adopted multiple guidance, strengthened psychological counseling, increased patients’ knowledge of their own health situation, and effectively alleviated patients’ depression and anxiety. All these results would be beneficial to the improvement of clinical outcomes. The present findings also demonstrated that the complication cases in the EG group were lower than those in the CG group (Table 3). FNAN improves patient satisfaction and their clinical outcomes. FNAN has strong pertinence and operability and is more associated with the theoretical model of internet nursing. Network nursing makes it possible to explore the nursing process and thus opens up a unknown cosmos of nursing professional to increase nursing knowledge.^[17]^ This study provided systematic and comprehensive evaluation guidance for the nursing care of mental health disorders in CHD patients. The patient's ineffective responses were judged on time, and the corresponding countermeasures could be performed to improve family nursing effectiveness immediately.

FNAN expands family nursing care of depression and anxiety, and promotes lung function rehabilitation in the CHD patients underwent CABG. There is growing evidence that family nursing conversations are beneficial to the subjects suffering from heart disease and to nurses.^[34]^ However, family nursing may be still limited with available knowledge for individuals. Psychoeducation has been reported to improve mental health in CHD patients by reducing Goldberg mental health questionnaire (GHQ) scores without reporting the effects of psychoeducation the sleep quality or lung function of the patients.^[35]^ Although the network has been used in the health care of CHD patients, important longitudinal outcomes information is still absent, and simply connecting current data sets will not solve the problem of CHD patients.^[36]^

There were also some shortcomings in FNAN. When applying FNAN, the following points should be noted:

(1) the application of FNAN intervention was relatively complicated, requiring the nursing staff to have rich nursing knowledge.
(2) The intervention mainly interfered with the existing ineffective response of the patient, and ignored the impact of the potential effective response on the patient because the patients paid more attention to their problems.

Although the intervention was insufficient sometimes, it also provided systematic theoretical guidance for nursing practice. In the application process, we should make full use of its advantages, reduce the impact of deficiency, and combine it with the overall modern nursing concept to improve the quality of care for CHD patients.

There were some limitations to the present work. The present study design is a single-center prospective clinical trial with a small sample and short intervention time, which leads to no significant difference in the observation indicators. Larger multicentric prospective studies are highly needed to better comprehend the potential protective role of FNAN on the clinical outcomes of heart failure patients. Validation of FNAN in multicentric prospective studies is warranted to generate sufficient data to formulate guidelines for improving its precision application in the CHD patients underwent CABG. If conditions permit, the number of samples should be increased in the clinic, and multi-center network, large-sample, prospective studies will be conducted to provide a more scientific theoretical basis for exploring the improvement of the mental health disorders, sleep quality and lung function parameters via FNAN intervention.

## 5 Conclusions

When compared with the common family nursing, FNAN intervention attenuated the severity of the depression and anxiety by reducing the scores of HAMA and HAMD. FNAN intervention improved the sleep quality by reducing the scores of PSQI. FNAN care improved lung function parameters of CHD patients underwent CABG by affecting the values of MVV, PaO2, SpO2, FEV1, and FVC and reduce the incidence of concomitant complications. Therefore, FNAN may be a potential approach in the improvement of clinical outcomes of CHD patients underwent CABG.

## Author contributions

**Clinical studies**: Lihua Huang, Haixia Zhang.

**Conceptualization:** Liying Jin, Ruijin Pan, Lihua Huang, Haixia Zhang, Mi Jiang, Hao Zhao.

**Data curation:** Liying Jin, Ruijin Pan, Lihua Huang, Haixia Zhang.

**Definition of intellectual content**: Mi Jiang.

**Experimental studies**: Ruijin Pan, Lihua Huang.

**Formal analysis:** Liying Jin, Ruijin Pan, Lihua Huang, Haixia Zhang.

**Funding acquisition:** Mi Jiang, Hao Zhao.

**Guarantor of integrity of the entire study**: Liying Jin.

**Investigation:** Liying Jin, Ruijin Pan, Lihua Huang, Haixia Zhang, Mi Jiang.

**Literature research**: Hao Zhao.

**Manuscript editing**: Mi Jiang.

**Manuscript preparation**: Mi Jiang.

**Manuscript review**: Hao Zhao.

**Methodology:** Liying Jin, Ruijin Pan, Lihua Huang, Haixia Zhang, Mi Jiang, Hao Zhao.

**Project administration:** Mi Jiang.

**Resources:** Lihua Huang, Mi Jiang.

**Software:** Liying Jin, Lihua Huang, Haixia Zhang.

**Statistical analysis**: Ruijin Pan, Lihua Huang.

**Study concepts**: Ruijin Pan.

**Study design**: Lihua Huang, Haixia Zhang.

**Supervision:** Ruijin Pan, Mi Jiang.

**Visualization:** Ruijin Pan.

**Writing – original draft:** Liying Jin, Ruijin Pan, Lihua Huang, Haixia Zhang.

**Writing – review & editing:** Mi Jiang, Hao Zhao.

## Supplementary Material

Supplemental Digital Content

## Supplementary Material

Supplemental Digital Content

## Supplementary Material

Supplemental Digital Content
